# PC6 injection enhanced the preventive effect against postoperative nausea of granisetron compared with intravenous injection in gynecological laparoscopic patients: a randomized controlled study

**DOI:** 10.3389/fphar.2026.1840347

**Published:** 2026-10-26

**Authors:** Jianying Song, Jia Tang, Juan Gu, Wanli Tian, Jin Zhou

**Affiliations:** 1 Department of Anesthesiology, West China Second University Hospital, Sichuan University, Chengdu, China; 2 Key Laboratory of Birth Defects and Related Diseases of Women and Children (Sichuan University), Ministry of Education, Chengdu, China

**Keywords:** acupoint, nausea, PC6, PONV, vomiting

## Abstract

**Background:**

Moderate to severe postoperative nausea and vomiting significantly impedes recovery and is inadequately managed by current pharmacotherapy. This study investigates whether granisetron injection at the PC6 acupoint enhances prevention of postoperative moderate to severe postoperative nausea and vomiting compared to intravenous injection.

**Methods:**

244 participants undergoing elective gynecologic endoscopic surgery under general anesthesia were randomly assigned to PC6 granisetron group, IV granisetron group, PC6 saline group and control group. All patients receive 5 mg dexamethasone during anesthesia induction. At the end of surgery, 2 mg granisetron was injected at the PC6 acupoint in PC6 granisetron group, 2 mg granisetron was injected intravenously in IV granisetron group, 2 ml saline was injected at the PC6 acupoint in PC6 saline group, no additional measures to prevent postoperative nausea and vomiting in control group.

**Results:**

The incidence of moderate-to-severe postoperative nausea in PC6 granisetron group was significantly lower than that in IV granisetron group, PC6 saline group and control group within 24 h after surgery. (14.8% [9/61] vs. 32.8% [20/61]; RR = 0.45, [95% CI: 0.22, 0.91]; P = 0.02 (14.8% [9/61] vs. 29.5% [18/61]; RR = 0.50 [95% CI: 0.24, 1.02]; P = 0.058, (14.8% [9/61] vs. 43.9% [25/57]; RR = 0.34 [95% CI: 0.17, 0.66]; P < 0.01.) These differences started from 2 h after surgery. There was no adverse events about PC6 acupoint injection was found.

**Conclusion:**

Granisetron injection at the PC6 acupoint enhances prevention of moderate-to-severe postoperative nausea within 24 h after surgery in patients undergoing elective gynecologic laparoscopic surgery under general anesthesia.

**Clinical Trial Registration:**

This study was registered at http://www.chictr.org.cn (ChiCTR2400085300).

## Introduction

Postoperative nausea and vomiting (PONV) continues to be a frequently encountered complication after surgery (Ao et al., 2025; Apfel et al., 2012; Chen et al., 2024a). Despite significant progress in contemporary anesthetic methods and antiemetic agents, PONV still markedly influences patients’ recovery and quality of life following an operation. Research indicates that the overall rate of PONV is about 20%–30% in general surgical cohorts, yet it may reach up to 80% among high-risk individuals—for example, women, nonsmokers, and those with a prior history of motion sickness (Gan et al., 2020). Aside from reducing patient comfort, PONV can provoke serious sequelae such as dehydration, electrolyte imbalances, and even wound separation (Pankiv et al., 2023; Deng et al., 2021).

Although management guidelines for PONV have been developed in recent years, its occurrence remains elevated, especially for moderate-to-severe postoperative nausea (Gan et al., 2020). Worries about adverse effects and the restricted effectiveness of standard antiemetic medications have heightened interest in non-drug treatments (Ongel et al., 2023). The Neiguan (PC6) acupoint, situated on the ventral side of the wrist between the tendons of the palmaris longus and flexor carpi radials (about three finger-widths above the wrist crease), has been demonstrated in both traditional Chinese medicine and modern investigations to aid in preventing PONV, particularly for relieving postoperative nausea (Yan et al., 2023). Consequently, the Fourth Consensus Guidelines for the Management of Postoperative Nausea and Vomiting recognize PC6 stimulation as a complementary and alternative therapy for PONV (Gan et al., 2020).

Several techniques for acupoint stimulation exist, including manual massage and acupuncture; nevertheless, these approaches can be demanding in time and effort (Ravi et al., 2024; Chen et al., 2024b; Chuang et al., 2025). Acupoint injection—a method in which a medicinal solution is introduced into an acupoint or a defined area—provides a more straightforward and convenient option. Based on meridian theory, this strategy merges the actions of the meridian, the acupoint, and the pharmacologic substance, thereby substantially improving therapeutic outcomes. Hence, it has emerged as a leading technique for acupoint stimulation (Wang and Kain, 2002; Yu et al., 2019).

According to the Fourth Consensus Guidelines, intravenous dexamethasone combined with granisetron serves as a foundational regimen for PONV prevention. This leads to a pertinent clinical question: would the combination of intravenous dexamethasone and PC6 acupoint injection of granisetron yield a better prophylactic effect against PONV relative to intravenous delivery of both agents alone?

Therefore, the objective of this study is to determine whether the preventive efficacy of intravenous dexamethasone plus PC6 acupoint injection of granisetron on moderate-to-severe PONV exceeds that of intravenous administration of the two drugs, and to investigate the possible mechanisms involved.

## Methods and materials

### Study design and ethics statements

This prospective, randomized controlled, single-center study evaluated the effect of dexamethasone combined with PC6 acupoint injection of granisetron on PONV. The study was reviewed and approved by the Research Ethics Committee of West China Second University Hospital, Sichuan University (Approval No. 2024046; Chair, Prof. Han Huang) on 8 April 2024. It was registered at the Chinese Clinical Trial Registry (http://www.chictr.org.cn; Registration No. ChiCTR2400085300; Principal Investigator: Jianying Song; Registration date: 4 June 2024). The study was conducted in accordance with the principles of the Declaration of Helsinki. All participants were capable of providing informed consent, and written informed consent was obtained from each participant before surgery.

This was a prospective, single-center, randomized, controlled trial. The primary objective was to compare the incidence of moderate-to-severe nausea within 24 h after surgery between patients receiving PC6 acupoint injection of granisetron plus intravenous dexamethasone and those receiving intravenous granisetron plus intravenous dexamethasone. The trial was designed as an exploratory superiority study. Two additional arms—PC6 saline injection (sham acupoint control) and no additional prophylaxis (control)—were included to explore the effect of acupoint mechanical stimulation and the background PONV rate, respectively.

### Setting and study population

The study enrolled women aged 18–50 years with American Society of Anesthesiologists (ASA) physical status I–II who provided informed consent for elective gynecologic endoscopic surgery under general anesthesia at our hospital between July 2024 and December 2024. Exclusion criteria were: (1) known allergy to granisetron or dexamethasone; (2) long-term use of long-acting antiemetics, emetic drugs, opioids, or glucocorticoids; (3) pregnancy or lactation; (4) developmental delay, mental disorders, or severe neurological diseases; (5) other conditions that could cause nausea or vomiting (e.g., hepatitis, peptic ulcer); (6) eczema or infection at the acupoint site; (7) refusal to participate in the study procedures.

### Acupoint localization and injection procedure

The PC6 (Neiguan) acupoint was identified according to standardized anatomical landmarks: on the volar aspect of the forearm, approximately 6.6 cm (three finger-breadths of the participant’s own fingers) proximal to the distal wrist crease, between the tendons of the palmaris longus and flexor carpi radialis muscles (Figure 1). To ensure blinding and avoid interference with the intravenous line, the injection was consistently performed on the non-dominant forearm contralateral to the IV access side. Participants were in the supine position with the forearm in neutral supination.

**FIGURE 1:**
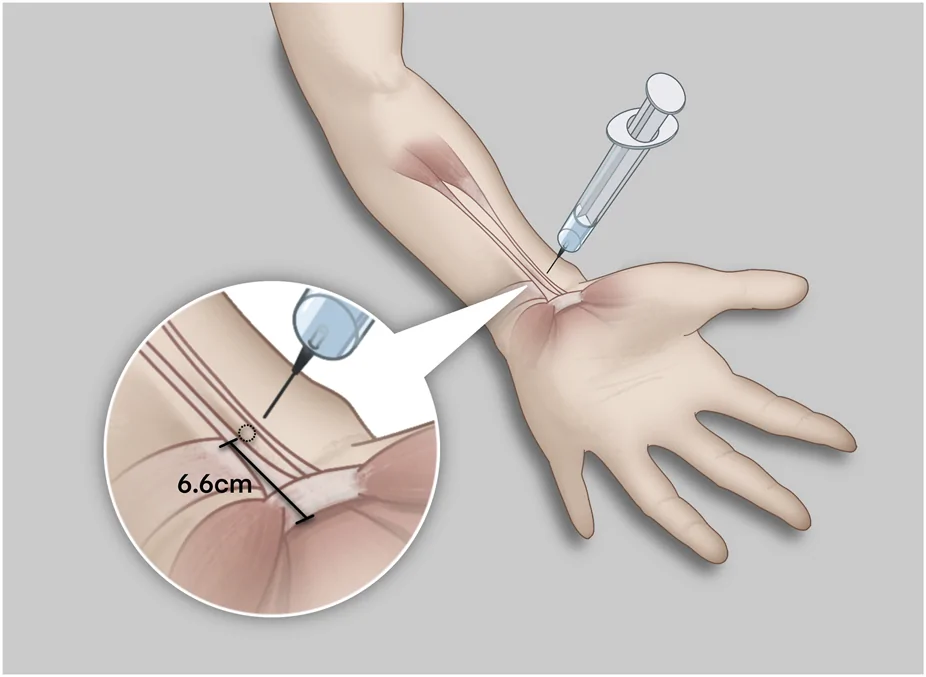
Schematic diagram of PC6 injection.

The skin was disinfected with iodophor in a circular motion over a 5-cm diameter area. A 5-mL syringe fitted with a 25-G (0.5 × 25 mm) needle was used to draw 2 mL of granisetron injection (1 mg/mL, total 2 mg). The needle was inserted perpendicularly to the skin to a depth of approximately 2 cm (adjusted for subcutaneous tissue thickness). Before drug administration, the plunger was gently aspirated to confirm absence of blood return. Granisetron was then injected slowly over 10–15 s. The needle was withdrawn immediately after injection, and the puncture site was compressed with a sterile dry cotton swab for ≥1 min to prevent local hematoma. No massage was applied after injection.

In the PC6 saline group, an identical volume (2 mL) of 0.9% normal saline was injected using the same anatomical localization, needle type, depth, and speed by the same investigator, serving as a sham acupoint control. In both groups, the injection was performed by an anesthesiologist who had received standardized training in PC6 localization and acupoint injection technique prior to the trial.

### Anesthesia and perioperative management

Patients were admitted to the operating room after standard preoperative fasting. Standard monitoring included pulse oximetry, heart rate, electrocardiography, non-invasive blood pressure, and invasive arterial blood pressure when indicated. Following pre-oxygenation via face mask, anesthesia was induced with midazolam (0.04 mg kg^-1^), propofol (1.5 mg kg^-1^), sufentanil (0.3 μg kg^-1^), and cisatracurium (0.2 mg kg^-1^). Intravenous dexamethasone (5 mg) was administered during induction. Mechanical ventilation was adjusted to maintain an end-tidal carbon dioxide (EtCO_2_) level of 35–45 mmHg. Anesthesia was maintained with sevoflurane (2%–3%) inhaled continuously, supplemented intermittently with sufentanil and cisatracurium to maintain anesthetic depth and to keep blood pressure within ±20% of baseline values. For postoperative analgesia, tramadol (2 mg kg^-1^) was administered during surgical closure. If residual neuromuscular blockade was present, neostigmine (1 mg) and atropine (0.5 mg) were administered intravenously for reversal. Following confirmation of adequate spontaneous respiration and recovery of consciousness, tracheal extubation was performed. Postoperative analgesia was provided either by a transversus abdominis plane (TAP) block (containing 5 mg dexamethasone and 40 mL of 0.25% ropivacaine) or by intravenous patient-controlled analgesia with sufentanil and tramadol, based on clinical assessment. Patients were then transferred to the post-anesthesia care unit (PACU) for monitoring. Throughout the perioperative period, episodes of hypotension, hypertension, bradycardia, tachycardia, or oxygen desaturation were managed at the discretion of the attending anesthesiologist.

### Timing of study interventions

All study interventions were administered after skin closure and before tracheal extubation, while the patient remained under general anesthesia and was fully unresponsive. Intravenous dexamethasone (5 mg) had been given as a fixed antiemetic backbone at anesthetic induction. The timing was standardized to ensure that the pharmacokinetic window of granisetron (or saline) was consistent across groups and that participants could not perceive the injection site or route during emergence. At the completion of surgery and before emergence, participants were allocated to one of four interventions according to the opened randomization envelope: (1) PC6 granisetron group​ – 2 mg granisetron (2 mL) injected at the PC6 acupoint as described above; (2) IV granisetron group​ – 2 mg granisetron (2 mL) injected intravenously via the existing IV line over 30 s; (3) PC6 saline group – 2 mL 0.9% saline injected at the PC6 acupoint using the identical technique; (4) control group​ – no additional antiemetic prophylaxis beyond the intraoperative dexamethasone (5 mg) and standard analgesia. All injections were performed by investigators who were not involved in outcome assessment or postoperative care.

### Rescue antiemetic protocol and postoperative PONV management

A standardized rescue antiemetic protocol was pre-specified in the trial protocol. In the post-anesthesia care unit (PACU) and operating room, any episode of sustained vomiting, retching was treated with a fixed rescue dose of intravenous granisetron 3 mg. Rescue medication was administered by the attending anesthesiologist or PACU nurse, who were blinded to group allocation but followed the written protocol. After transfer to the surgical ward, if a patient reported vomiting, retching, or nausea (VAS ≥7) unrelieved by conservative measures (e.g., repositioning, oral hydration), rescue antiemetics (IV granisetron 3 mg or alternative agents at the ward physician’s discretion) were administered and the drug name, dose, and time were recorded. All rescue antiemetic administrations were documented as a secondary outcome and adjudicated by an independent assessor blinded to group allocation.

## Randomization and blinding

Eligible participants were randomly allocated in a 1:1 ratio to one of four groups: the PC6 granisetron group, the IV granisetron group, the PC6 saline group, or the control group. The randomization sequence was generated by an independent investigator using Excel 2019 (Microsoft) and concealed in sequentially numbered, sealed, opaque envelopes. Study coordinators (who were not involved in follow-up assessments) screened potential participants from surgical schedules. After receiving information about the study purpose and procedures, interested patients provided written informed consent and were assigned a participant number. Following surgery, the envelope corresponding to the participant number was opened to reveal the group assignment. All interventions were administered by researchers who did not participate in subsequent follow-up or treatment. Outcome assessors, who were blinded to group assignment and the specific intervention performed, collected all study data. Since all interventions took place before emergence from anesthesia, participants remained unaware of their group allocation.

### Outcome assessments

The primary outcome was the incidence of moderate-to-severe nausea within the first 24 h after surgery. Nausea was defined as a subjective feeling of stomach discomfort accompanied by an urge to vomit. Nausea severity was assessed using a 0–10 visual analog scale (VAS; 0 = no nausea, 10 = worst possible nausea). A score of ≥4 was defined as moderate-to-severe nausea, a threshold consistent with previous PONV and nausea literature (Gan et al., 2020; Wu et al., 2023). Secondary outcomes included: the incidence of moderate-to-severe nausea at other time points; the overall incidence of nausea and vomiting; time to hospital discharge; and the requirement for rescue antiemetic medication. Vomiting was defined as sustained, forceful contraction of the abdominal muscles with or without expulsion of gastric contents (Ismail et al., 2019; Bi et al., 2022).Time to discharge was calculated as the interval from the day of surgery to the day of leaving the hospital. Adverse events included skin infection, redness, or other local reactions at the PC6 injection site within 24 h postoperatively. The 24-h postoperative morphine equivalent dose was recorded (conversion factors: 1 mg morphine = 10 mg tramadol = 0.2 mg butorphanol = 1 mg dezocine = 0.5 mg oxycodone = 1 µg sufentanil; all intravenous). The Apfel score was used to predict PONV risk (Pierre et al., 2002). Trained assessors, blinded to group allocation, conducted follow-up evaluations at 2, 6, 12, and 24 h after surgery to record episodes of nausea and vomiting and any adverse events.

### Statistical analysis

Sample size was calculated for the primary comparison (PC6 granisetron vs. IV granisetron) based on preliminary data estimating a 24-h moderate-to-severe nausea incidence of 16% in the PC6 group and 29% in the IV group. With a two-sided α = 0.05 and 80% power, 56 patients per group were required; 61 per group were enrolled to allow for a 10% dropout rate. The PC6 saline and control groups were allocated equal numbers for balanced randomization and exploratory comparison; the trial was not powered for pairwise comparisons involving these two arms. Analysis followed a modified intention-to-treat principle (all randomized patients except 4 in the control group who received protocol-violating 10 mg dexamethasone were analyzed).

Categorical variables are presented as n (%) and compared using Pearson’s χ^2^ or Fisher’s exact test. Continuous variables are presented as mean ± SD or median (IQR) as appropriate. Relative risks (RR) with 95% confidence intervals (CI) were calculated for binary outcomes. PC6 granisetron vs. IV granisetron at 24 h was the pre-specified primary comparison.​ All other between-group pairwise comparisons and multiple time-point secondary analyses were considered exploratory; no adjustment for multiplicity was applied, and inferences were drawn conservatively based on 95% CIs crossing or excluding 1. A two-tailed P < 0.05 was reported for the primary comparison; for exploratory comparisons, exact P values are provided without multiplicity correction. Analyses were performed using SPSS 22.0.

## Results

### Patient characteristics

Between July and December 2024, 264 patients were screened for eligibility. Twenty did not meet the inclusion/exclusion criteria (13 failed to meet inclusion criteria, 7 declined to participate). Consequently, 244 participants were randomized to the PC6 granisetron group (n = 61), the IV granisetron group (n = 61), the PC6 saline group (n = 61), and the control group (n = 61). In the control group, four participants inadvertently received 10 mg of dexamethasone and were therefore excluded from the final analysis (Figure 2).

**FIGURE 2:**
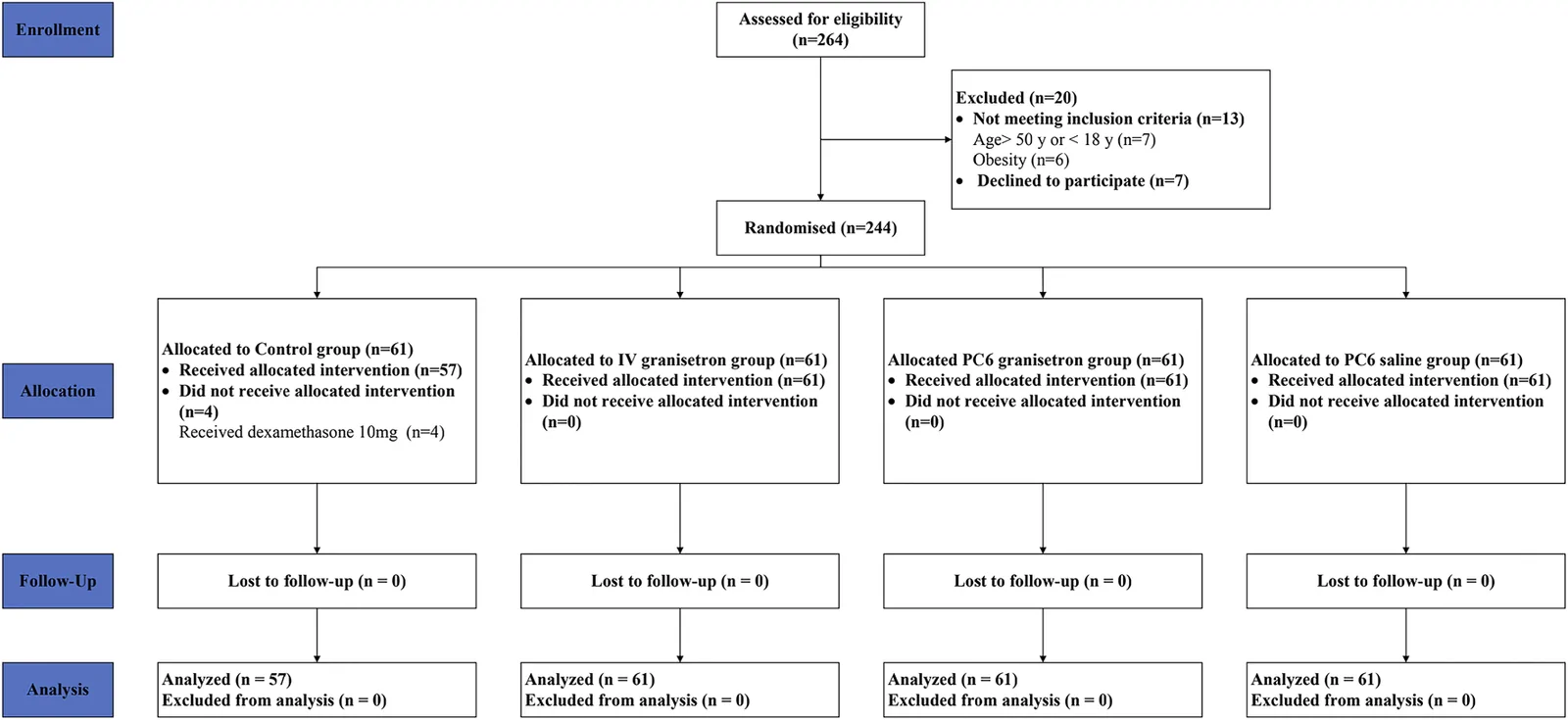
Flow chart.

A total of 179 patients (74.6%) had an Apfel risk score of 3–4. There were no significant differences among the groups in baseline characteristics, Apfel score, preoperative fasting time, duration of anesthesia, intraoperative fluid intake, blood loss, or postoperative analgesic consumption (Table 1).

**TABLE 1:** Baseline characteristics of the four groups.

Index	Control (N = 57) Mean ± SD	IV granisetron (N = 61) Mean ± SD	PC6 granisetron (N = 61) Mean ± SD	PC6 saline (N = 61) Mean ± SD	P Value
Age (years)	37.65 ± 7.48	35.49 ± 8.41	36.82 ± 7.92	34.11 ± 8.12	0.09
BMI (kg/m^2^)	21.95 ± 2.46	21.47 ± 2.56	21.63 ± 2.52	21.85 ± 2.47	0.72
Preoperative fasting time (h)	11.53 ± 2.94	11.38 ± 2.40	12.15 ± 2.72	11.49 ± 2.99	0.41
Duration of anesthesia (min)	145.16 ± 47.95	155.05 ± 55.21	144.34 ± 38.59	133.57 ± 37.76	0.08
Duration of surgery (min)	126.46 ± 45.73	136.57 ± 55.81	125.43 ± 39.55	113.9 ± 36.03	0.05
Intraoperative blood loss (ml)	49.7 ± 84.39	53.85 ± 60.43	53.2 ± 73.49	27.57 ± 21.88	0.08
Intraoperative infusion volume (L)	1.27 ± 0.38	1.38 ± 0.41	1.25 ± 0.33	1.30 ± 0.39	0.24
Type of surgery: n (%)					
Hysterectomy	14 (24.6%)	14 (23.0%)	13 (21.3%)	6 (9.8%)	0.16
Excision of cyst	19 (33.3%)	20 (32.8%)	22 (36.1%)	24 (39.3%)	0.87
Myoma enucleation	14 (24.6%)	13 (22.8%)	8 (14.0%)	13 (22.8%)	0.51
Others	10 (17.5%)	14 (23.0%)	18 (29.5%)	18 (29.5%)	0.37
Apfel score for the risk of PONV: n (%)					
1	1 (1.8%)	0 (0.0%)	0 (0.0%)	1 (1.6%)	0.55
2	11 (19.3%)	20 (32.8%)	14 (23.0%)	14 (23.0%)	0.36
3	36 (63.2%)	32 (52.5%)	37 (60.7%)	37 (60.7%)	0.65
4	9 (16.4%)	9 (14.8%)	10 (16.4%)	9 (14.8%)	0.99
TAP, n (%)	45 (78.9%)	50 (82.0%)	54 (88.5%)	54 (88.5%)	0.37
PCA, n (%)	19 (33.3%)	16 (26.2%)	19 (31.1%)	13 (21.3%)	0.47

Data are presented as means ± standard or number (%), PONV: postoperative nausea and vomiting, Apfel score for the risk of PONV(0-4): Female, non-smoker, history of PONV, or motion sickness and post-operative use of opioids; TAP, transverse abdominal muscle plane block used for postoperative analgesia; PCA, patient controlled analgesia used for postoperative analgesia.

### Primary outcome

The number of patients with a nausea score ≥4 (moderate-to-severe nausea) within 24 h after surgery was significantly lower in the PC6 granisetron group than in the IV granisetron group, the PC6 saline group, and the control group (Table 2). PC6 granisetron vs. IV granisetron: 14.8% (9/61) vs. 32.8% (20/61); RR = 0.45, 95% CI: 0.22–0.91; P = 0.02. PC6 granisetron vs. PC6 saline: 14.8% (9/61) vs. 29.5% (18/61); RR = 0.50, 95% CI: 0.24–1.02; P = 0.058. PC6 granisetron vs. control: 14.8% (9/61) vs. 43.9% (25/57); RR = 0.34, 95% CI: 0.17–0.66; P < 0.01. No significant difference was observed among the other three groups (Table 3).

**TABLE 2:** Comparison of the incidence of PONV between the four groups.

Index	Control (N = 57)	IV granisetron (N = 61)	PC6 granisetron (N = 61)	PC6 saline (N = 61)
Incidence of VAS of nausea≥4, n (%)				
0-2 h	12 (21.1)^a^	5 (8.2%)^b^	0 (0.0)^c^	8 (13.1)^a,b^
0-6 h	18 (31.6)^a^	10 (16.4)^a^	3 (4.9)^b^	11 (18.0)^a^
0-12 h	22 (38.6)^a^	16 (26.2)^a^	6 (9.8)^b^	16 (26.2)^a^
0-24 h	25 (43.9)^a^	20 (32.8)^a^	9 (14.8)^b^	18 (29.5)^a,b^
Incidence of nausea, n (%)				
0–2 h	14 (24.6)^a^	7 (11.5)^a,b^	2 (3.3)^b^	13 (21.3)^a^
0-6 h	21 (36.8)^a^	16 (26.2)^a,b^	11 (18.0)^b^	18 (29.5)^a,b^
0-12 h	25 (43.9)^a^	22 (36.1)^a,b^	16 (26.2)^b^	25 (41.0)^a,b^
0-24 h	28 (49.1)^a^	27 (44.3)^a^	22 (36.1)^a^	26 (42.6)^a^
Incidence of vomiting, n (%)				
0–2 h	7 (12.3)^a^	1 (1.6)^b^	1 (1.6)^b^	3 (4.9)^a,b^
0-6 h	12 (21.1)^a^	4 (6.6)^b^	4 (6.6)^b^	6 (9.8)^a,b^
0–12 h	19 (33.3)^a^	12 (19.7)^a,b^	9 (14.8)^b^	17 (27.9)^a,b^
0-24 h	24 (42.1)^a^	12 (19.7)^b^	10 (16.4)^b^	18 (29.5)^ab^
*VAS of nausea				
2 h	5.8 ± 2.4^a^	4.5 ± 2.5^a^	(2.0, 3.0)^a^	4.5 ± 2.6^a^
6 h	5.3 ± 2.1^a^	4.2 ± 1.8^a,b^	3.3 ± 1.8^b^	4.1 ± 1.9^a,b^
12 h	5.1 ± 2.4^a^	5.4 ± 2.6^a^	4.3 ± 2.4^a^	5.4 ± 2.9^a^
24 h	5.6 ± 1.8^a^	4.8 ± 1.8^a^	3.6 ± 2.8^a^	4.6 ± 2.8^a^

Data are presented as means ± standard or number (%), PONV: postoperative nausea and vomiting, VAS: visual analog scale (for nausea (0 = no nausea, 10 = most severe nausea; ≥4 = moderate to severe nausea), a, b, c: There has statistical difference among the groups with the different letters (P < 0.05), * Average VAS, for each patient who had nausea.

**TABLE 3:** Comparison of the incidence of PONV between the IV granisetron group and the PC6 granisetron group.

Index	PC6 granisetron (N = 61)	IV granisetron (N = 61)	Relative risk (95%CI)	P Value
Incidence of VAS of nausea ≥4, (n, %)				
0–2 h	0 (0.0%)	5 (8.2%)	/	0.06
0-6 h	3 (4.9%)	10 (16.4%)	0.26 (0.07-1.00)	0.04
0-12 h	6 (9.8%)	16 (26.2%)	0.30 (0.11-0.83)	0.02
0-24 h	9 (14.8%)	20 (32.8%)	0.36 (0.15-0.83)	0.02
Incidence of nausea (n, %)				
0–2 h	2 (3.3%)	7 (11.5%)	0.26 (0.05-1.25)	0.16
0-6 h	11 (18.0%)	16 (26.2%)	0.63 (0.03-1.43)	0.27
0-12 h	16 (26.2%)	22 (36.1%)	0.63 (0.29-1.43)	0.24
0-24 h	22 (36.1%)	27 (44.3%)	0.71 (0.34-1.43)	0.36
Incidence of vomiting (n, %)				
0–2 h	1 (1.6%)	1 (1.6%)	1.0 (0.06-1.67)	1.00
0-6 h	4 (6.6%)	9 (9.8%)	0.67 (0.17-2.00)	0.51
0–12 h	9 (14.8%)	12 (19.7%)	0.71 (0.27-2.00)	0.47
0-24 h	10 (16.4%)	12 (19.7%)	0.83 (0.31-2.00)	0.64
Use of rescue medicine (n, %)	3 (4.9%)	3 (4.9%)	1.00 (0.19-5.26)	1.00
Discharge time (days)	3.02 ± 1.06	3.21 ± 2.24	/	0.54
Postoperative morphine equivalent dose (mg)	57.2 ± 1.35	57.3 ± 1.46	/	0.82

Data are presented as means ± standard or number (%), PONV: postoperative nausea and vomiting, VAS, visual analog scale (for nausea (0 = no nausea, 10 = most severe nausea; ≥4 = moderate to severe nausea).

### Secondary outcomes

The incidence of moderate-to-severe nausea in the PC6 granisetron group was significantly lower than in the other three groups at all time points from 2 to 24 h after surgery (Figure 3). The IV granisetron group showed a lower incidence than the control group only within the first 2 h.

**FIGURE 3:**
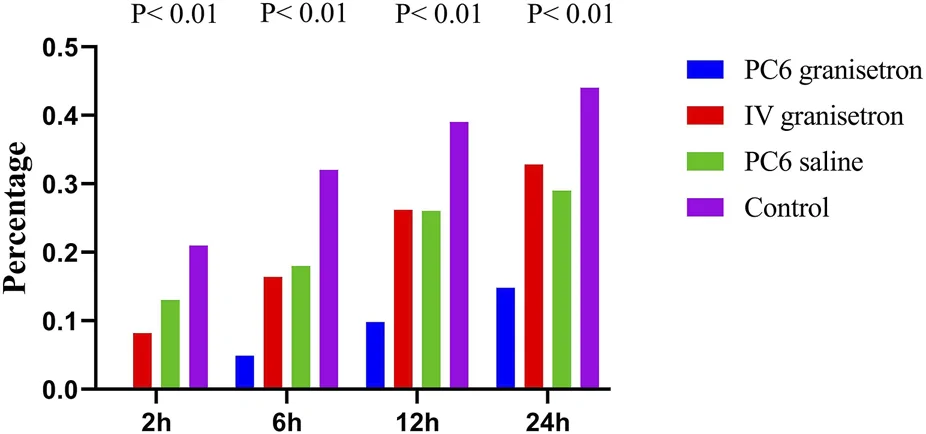
Postoperative incidence of VAS of nausea ≥4 from 2 to 24 h in the four groups. VAS, visual analog scale for nausea (0 = no nausea, 10 = most severe nausea; ≥4 = moderate to severe nausea).

The overall incidence of nausea in the PC6 granisetron group was significantly lower than in the control group from 2 to 12 h postoperatively. The frequency of vomiting within 24 h was lower in both the IV granisetron and PC6 granisetron groups compared with the control group, with no significant difference between the two active treatment groups.

A total of six patients in the control group, 3 in the PC6 granisetron group, 3 in the IV granisetron group, and 3 in the PC6 saline group required rescue antiemetics in the ward; these differences were not statistically significant. There were no significant differences among the four groups in time to discharge or 24-h postoperative morphine equivalent dose (Table 2; Table 3).

### Subgroup analysis

In the subgroup of patients with an Apfel risk score of 3–4, the number of patients with a nausea score ≥4 was significantly lower in the PC6 granisetron group than in the other three groups (P < 0.01).

### Adverse events

No complications—such as swelling, pain, infection, or skin damage at or around the PC6 injection site—were observed following PC6 injection. No other adverse events were reported by any participant.

## Discussion

This single-center, double-blind, randomized controlled trial assessed the prophylactic efficacy of granisetron delivered via PC6 acupoint injection versus the intravenous route for preventing postoperative nausea and vomiting (PONV) in patients receiving elective gynecological laparoscopic surgery under general anesthesia. The group receiving PC6 acupoint injection demonstrated a significantly lower incidence of moderate-to-severe postoperative nausea compared to the intravenous injection group. Furthermore, the anti-nausea effect provided by PC6 acupoint injection of granisetron was more sustained. No adverse events attributable to the treatment were noted. These observations align with prior literature supporting PC6 acupoint stimulation as a valuable complementary and alternative approach for managing PONV (Gan et al., 2020; Yan et al., 2023).

The approach of PC6 acupoint injection seems to integrate or potentiate the actions of both the pharmacological agent and the acupoint stimulation, given that its preventive performance surpassed that of intravenous granisetron alone and of saline injection at the PC6 site. Although the incidence of moderate-to-severe nausea in the PC6 granisetron group was numerically half that of the PC6 saline group (14.8% vs. 29.5%), the 95% CI for this comparison (RR 0.50, 95% CI 0.24–1.02) crossed the null value, and the exact P value was 0.058. This reflects the fact that the study was powered only for the primary comparison (PC6 granisetron vs. IV granisetron) and was underpowered for the saline control comparison. Therefore, we do not claim a statistically significant incremental benefit of granisetron over saline injection at PC6; rather, the data suggest a possible drug–acupoint synergistic trend that warrants larger confirmatory trials. The overall results indicate that combining PC6 acupoint injection of granisetron with intravenous dexamethasone presents a straightforward and effective strategy for PONV prophylaxis. This is particularly relevant for preventing moderate-to-severe nausea in the studied patient population. Further investigation through multicenter trials with larger cohorts is needed to verify the broader applicability and safety profile of this method.

In our study, the PONV incidence in the intravenous granisetron plus dexamethasone group was 32.8%. This figure is consistent with two large-scale cohort studies involving Chinese patients, which reported that even with a dual-prophylaxis regimen combining a 5-HT3 receptor antagonist and dexamethasone, PONV rates remain considerable, ranging from 31.7% to 48.7% (Pankiv et al., 2023; Huang et al.). The current trial primarily included patients undergoing gynecological surgery, a population known to be at high inherent risk for PONV; this likely explains why the observed incidence was relatively high compared to some previous studies (Weibel et al., 2021). Multiple variables related to the patient, anesthesia, and surgery can influence PONV, and these factors were adequately balanced among the four groups in our trial (Elvir-Lazo et al., 2020).

Nausea severity was assessed using a visual analog scale (VAS, score ≥4 defined as moderate-to-severe), an instrument adapted from the Pregnancy-Unique Quantification of Emesis score and the pain VAS (Wu et al., 2023). We found the incidence of moderate-to-severe nausea to be 32.8% in the intravenous granisetron plus dexamethasone group, compared to 14.8% in the group receiving PC6 acupoint injection of granisetron plus intravenous dexamethasone. Multiple clinical studies have shown that acupuncture or stimulation at specific acupoints, including Neiguan (PC6), Waiguan (SJ5), Zusanli (ST36), and Hegu (LI4), can effectively reduce PONV (Xiao et al., 2023; Yılmaz et al., 2023; Lin et al., 2016; Qi et al., 2014; Chen et al., 2021; Cheng et al., 2024; Chen et al., 2023; Pakiş Çetin and Çevik Kaya, 2024; Zheng et al., 2026). The PC6 acupoint, due to its accessible location on the wrist, is particularly convenient for intraoperative use. Compared to traditional acupoint stimulation methods like acupuncture, massage, or transcutaneous electrical nerve stimulation, acupoint injection offers a simpler and more practical alternative.

According to the principles of traditional Chinese medicine, the PC6 acupoint is considered a “transport” (shu) point capable of enhancing therapeutic agent efficacy. Therefore, administering granisetron via the PC6 acupoint may potentiate its preventive effect against PONV relative to systemic intravenous delivery. Our results confirm that PC6 acupoint injection of granisetron significantly reduced postoperative nausea severity compared to intravenous injection. We also noted that the beneficial effect of the PC6 injection persisted longer than that of the intravenous route when evaluating both against the control. The inclusion of a PC6 saline injection group was important to rule out the possibility that the observed benefit stemmed solely from the mechanical stimulation of the acupoint, though the sample size being underpowered to detect a statistically significant difference. Additionally, the PC6 acupoint injection method appeared safe, with no major complications reported. However, more extensive safety data are required.

Our study has several limitations. First, the generalizability of the findings is constrained by its single-center design and its restriction to female patients undergoing gynecological laparoscopic surgery. Future multicenter randomized controlled trials encompassing more diverse patient populations are necessary to thoroughly evaluate the effectiveness of this granisetron administration route for PONV prevention. Second, the reduction in PONV severity we observed may not be generalizable to patient groups with an inherently low baseline risk of PONV. Finally, while the synergy between the Neiguan point and granisetron can be interpreted through the lens of traditional Chinese medicine theory, further research is warranted to elucidate the underlying biological mechanisms from a modern biomedical perspective and to validate our results. Such work is needed to firmly establish PC6 acupoint injection of granisetron as a validated method for PONV prevention.

In conclusion, in patients undergoing elective gynecological laparoscopic surgery under general anesthesia, PC6 acupoint injection of granisetron yielded a greater reduction in PONV severity within the first 24 postoperative hours than intravenous injection, suggesting a combined or synergistic effect between the drug and PC6 stimulation. Large-scale clinical trials are essential to corroborate these findings before PC6 acupoint injection of granisetron can be advocated for routine clinical use in preventing PONV, either in gynecological laparoscopic surgery or in other surgical contexts.

## Data Availability

The original contributions presented in the study are included in the article/supplementary material, further inquiries can be directed to the corresponding author.
